# eIF3a Regulation of NHEJ Repair Protein Synthesis and Cellular Response to Ionizing Radiation

**DOI:** 10.3389/fcell.2020.00753

**Published:** 2020-08-19

**Authors:** Rima Tumia, Chao J. Wang, Tianhan Dong, Shijie Ma, Jenny Beebe, Juan Chen, Zizheng Dong, Jing-Yuan Liu, Jian-Ting Zhang

**Affiliations:** ^1^Department of Pharmacology and Toxicology, Indiana University School of Medicine, Indianapolis, IN, United States; ^2^Department of Cancer Biology, University of Toledo College of Medicine and Life Sciences, Toledo, OH, United States; ^3^Department of Medicine, University of Toledo College of Medicine and Life Sciences, Toledo, OH, United States

**Keywords:** eukaryotic initiation factor 3a (eIF3a), DNA repair, radiation, resistance, mRNA translation, protein synthesis, gamma-H2A histone family member X (γ-H2AX)

## Abstract

Translation initiation in protein synthesis regulated by eukaryotic initiation factors (eIFs) is a crucial step in controlling gene expression. eIF3a has been shown to regulate protein synthesis and cellular response to treatments by anticancer agents including cisplatin by regulating nucleotide excision repair. In this study, we tested the hypothesis that eIF3a regulates the synthesis of proteins important for the repair of double-strand DNA breaks induced by ionizing radiation (IR). We found that eIF3a upregulation sensitized cellular response to IR while its downregulation caused resistance to IR. eIF3a increases IR-induced DNA damages and decreases non-homologous end joining (NHEJ) activity by suppressing the synthesis of NHEJ repair proteins. Furthermore, analysis of existing patient database shows that eIF3a expression associates with better overall survival of breast, gastric, lung, and ovarian cancer patients. These findings together suggest that eIF3a plays an important role in cellular response to DNA-damaging treatments by regulating the synthesis of DNA repair proteins and, thus, eIIF3a likely contributes to the outcome of cancer patients treated with DNA-damaging strategies including IR.

## Introduction

Eukaryotic initiation factors (eIFs) are a family of proteins that play important roles in mRNA translation and protein synthesis. Recent growing evidence suggests that eIFs do not just participate in translation initiation of global mRNAs but may also regulate synthesis of a subset of proteins (Dong and Zhang, 2006; Dong et al., 2009). These regulatory functions have been thought to contribute to the potential oncogenic role of eIFs (Hershey, 2015). Indeed, many eIFs were found to have higher expression in human tumors and shown to have oncogenic activity (Yin et al., 2011a). One of these eIFs, eIF3a, has been found to overexpress in many human cancers including cancers of the breast (Bachmann et al., 1997), cervix (Dellas et al., 1998), esophagus (Chen and Burger, 1999), stomach (Chen and Burger, 2004), lung (Pincheira et al., 2001), and bladder (Spilka et al., 2014), and it was thought to be a proto-oncogene. Indeed, knocking down eIF3a expression reversed the malignant phenotype of human cancer cells (Dong et al., 2004) while overexpressing ectopic eIF3a transformed NIH3T3 fibroblast cells (Zhang et al., 2007) *in vitro*.

Interestingly, eIF3a overexpression resulted in cellular sensitivity to cisplatin by regulating nucleotide excision repair (NER) *via* suppressing the synthesis of NER proteins (Liu et al., 2011). It has also been shown that eIF3a upregulation increases cellular sensitivity to anticancer drug doxorubicin, which inhibits topoisomerase II and causes DNA double-strand breaks (DSBs; Yin et al., 2011b). Extensive DSBs induced by various exogenous and endogenous factors are one of the most fatal forms of DNA damages (Helleday et al., 2007; Reynolds et al., 2012) and are used for treating human cancers in the form of chemo and radiation therapy. However, cancer cells with efficient repair of DSBs are able to survive these treatments that cause DSBs using two major mechanisms of repair of DSBs, homologous recombination (HR) and non-homologous end joining (NHEJ; Hartlerode and Scully, 2009; Pardo et al., 2009). While HR repairs the damages using undamaged and symmetrical chromosome as a template during the S or G phase of the cell cycle (Helleday et al., 2007; Branzei and Foiani, 2008), NHEJ repairs DSBs throughout all cell cycle phases and is the major pathway in repairing ionizing radiation (IR)-induced DSBs (van Gent et al., 2001; Rothkamm et al., 2003; Lieber, 2010). The major proteins important in NHEJ repair of DSBs include Ku (Ku70, Ku80) and DNA-PKcs to form the DNA-PK enzyme (Gottlieb and Jackson, 1993; Yaneva et al., 1997; Mari et al., 2006; Uematsu et al., 2007).

In this study, we tested the hypothesis that eIF3a may regulate the cellular response to treatments that cause DSBs by regulating the synthesis of DSB repair proteins. We examined the role of eIF3a in the cellular response to IR because IR is a common and an important strategy for treating many types of human cancers (Hoeijmakers, 2001; Kastan and Bartek, 2004; Lobrich and Jeggo, 2007) and is known to cause DSBs. We found that eIF3a sensitized the cellular response to IR treatments by downregulating NHEJ repair *via* inhibiting the synthesis of NHEJ repair proteins including Ku70, Ku80, and DNA-PKcs. These findings suggest that translational regulation of gene expression and eIF3a play important roles in the cellular response to DNA-damaging treatments and, thus, may underline the molecular basis of their functions in cellular response to drug/radiation-induced DNA damages and in cancer prognosis.

## Results

### Role of Eukaryotic Initiation Factor 3a in the Cellular Response to Ionizing Radiation Treatment

**FIGURE 1:**
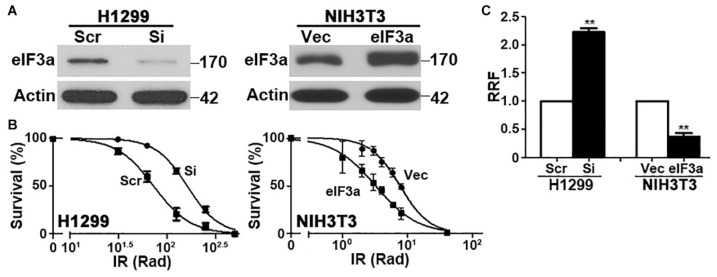
Effect of eukaryotic initiation factor (eIF)3a expression on the cellular response to ionizing radiation (IR). Western blot analyses **(A)** and colony formation assay following IR treatment **(B)** of H1299 cells with transient eIF3a knockdown and NIH3T3 cells with stable eIF3a overexpression compared with their respective control cells. Actin was used as a loading control. Panel **(C)** shows a summary of eIF3a effects on cellular sensitivity to IR treatments. Relative resistance factor (RRF) was derived by dividing the IC_50_ of the test cells by that of their control cells (*n* = 3, **P < 0.01).

To determine the potential role of eIF3a in the cellular response to IR, we first knocked down eIF3a expression using siRNA in H1299 cells, which have a high level of endogenous eIF3a (Figure 1A) followed by analysis of cellular response to IR using colony formation assay. As shown in Figures 1B,C, H1299 cells with eIF3a knockdown (H1299/Si) are significantly more resistant with a 2-fold increase in relative resistance factor (RRF) than the control H1299 cells transfected with scrambled control siRNA (H1299/Scr). To confirm this observaiton, we performed a reverse experiment by using the stable NIH3T3 cells with eIF3a overexpression (NIH3T3/eIF3a) (Figure 1A) and tested their response to IR in comparison with the control cells transfected with the empty vector (NIH3T3/Vec). As shown in Figures 1B,C, NIH3T3/eIF3a cells are remarkably more sensitive than the control NIH3T3/Vec cells to IR with ∼2-fold reduction in RRF. Thus, eIF3a expression may affect cellular sensitivity to IR treatments.

### Effect of Eukaryotic Initiation Factor 3a on Gamma-H2A Histone Family Member X Expression Following Ionizing Radiation Treatment

**FIGURE 2:**
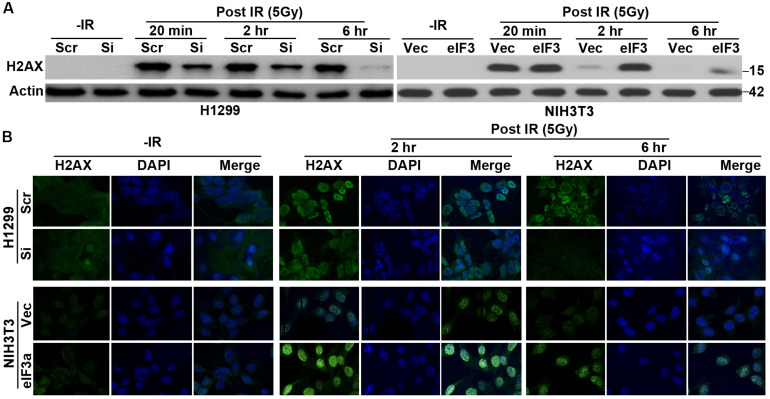
Effect of eukaryotic initiation factor (eIF)3a on ionizing radiation (IR)-induced gamma-H2A histone family member X (γ-H2AX). Western blot **(A)** and immunofluorescence staining **(B)** analyses of γ-H2AX in H1299 cells with transient eIF3a knockdown and in NIH3T3 cells with stable eIF3a overexpression compared with their respective control cells following IR treatments.

To investigate how eIF3a affects the cellular response to IR, we tested the hypothesis that eIF3a may regulate the repair of DSBs induced by IR. For this purpose, we first tested the effect of eIF3a on gamma-H2A histone family member X (γ-H2AX) expression, a marker for DSB (Kuo and Yang, 2008), following IR treatment. As shown in Figure 2A, little γ-H2AX was detected in either H1299/Si or the control H1299/Scr cells without IR treatments. However, the γ-H2AX level drastically increased following IR treatment in these cells at 20 min after IR treatment. Interestingly, at 6 h after IR, γ-H2AX in H1299/Si cells returned essentially to the basal level while it remained high in the control H1299/Scr cells. We also performed similar experiments using NIH3T3/eIF3a and the control NIH3T3/Vec cells and found that eIF3a overexpression clearly delayed DNA repair, as indicated by the delayed disappearance of γ-H2AX (Figure 2A), consistent with the findings using H1299/Si and H1299/Scr cells.

To verify the above findings, we performed immunofluorescence staining of γ-H2AX in the nuclei of these cells at 2 and 6 h after IR exposure. As shown in Figure 2B, the high level of punctate staining of γ-H2AX in the nuclei of H1299/Si cells observed at 2 h following IR disappeared at 6 h. However, the punctate staining in the control H1299/Scr cells remained high at 6 h following IR. Similarly, NIH3T3/eIF3a cells retained high levels of γ-H2AX, whereas the control NIH3T3/Vec cells lost γ-H2AX staining at 6 h following IR. These observations are consistent with the results shown using Western blot analysis. Thus, it is possible that eIF3a suppresses the repair of DSB induced by IR.

### Effect of Eukaryotic Initiation Factor 3a on DNA Damage Induced by Ionizing Radiation

**FIGURE 3:**
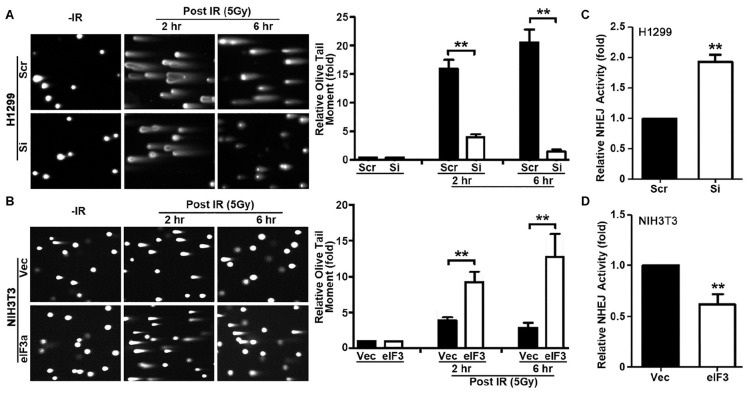
Role of eukaryotic initiation factor (eIF)3a in non-homologous end joining (NHEJ) repair of ionizing radiation (IR)-induced double-strand breaks (DSBs). **(A,B)** Comet assay was used to determine eIF3a effects on the level of DSBs induced by IR in H1299 cells with transient eIF3a knockdown **(A)** and NIH3T3 cells with stable eIF3a overexpression **(B)** compared with their respective control cells. The histograms show the summary of quantitative analysis of Olive tail moment in these cells. **(C,D)** Host cell reactivation assays using reporter constructs were performed using H1299 cells with eIF3a knockdown **(C)** and NIH3T3 cells with eIF3a stable overexpression **(D)** compared with their respective control cells (*n* = 3; **P < 0.01).

In the above studies, we used γ-H2AX as a DNA damage marker to evaluate DNA damage and repair. To directly evaluate DNA damage induced by IR in the presence of different levels of eIF3a, we performed neutral comet assay at 2 and 6 h following IR treatment. Cells at 20 min following IR were not tested using this assay because the short time was difficult to control for the comet assay. As shown in Figure 3A, H1299/Si cells clearly have significantly lower Olive tail moment than the control H1299/Scr cells at both 2 and 6 h following IR treatment. It is noteworthy that the relative Olive tail moment was reduced at 6 h compared with 2 h following IR in H1299/Si cells while it remained high in H1299/Scr cells, suggesting that little DSBs were repaired in H1299/Scr cells compared with H1299/Si cells. Similarly, NIH3T3/eIF3a cells had significantly higher Olive tail moment than its control NIH3T3/Vec cells (Figure 3B) following IR treatment, and less DSBs were repaired in NIH3T3/eIF3a than in the control NIH3T3/Vec cells as indicated by the change in the relative Olive tail moment between 2 and 6 h following IR. Thus, eIF3a likely inhibits the repair of DSBs induced by IR, and cells with high levels of eIF3a retain higher levels of DSBs following IR while cells with lower eIF3a retain lower level of DSBs.

### Role of Eukaryotic Initiation Factor 3a in Non-homologous End Joining Repair of Double-Strand Breaks

To determine if eIF3a regulates repairs of DSBs, we next performed host cell reactivation (HCR) assay of NHEJ activity since NHEJ is the main repair pathway of IR-induced DNA damages and it is independent of cell cycle stages as discused above. As shown in Figure 3C, H1299/Si cells had a 2-fold increase in NHEJ activity compared with the control H1299/Scr cells. Consistently, the NHEJ activity in NIH3T3/eIF3a cells was decreased by 40% compared with the control NIH3T3/Vec cells (Figure 3D). These findings suggest that eIF3a may play an important role in suppressing NHEJ repair of DSBs.

### Eukaryotic Initiation Factor 3a Regulates Synthesis of Non-homologous End Joining Repair Proteins

**FIGURE 4:**
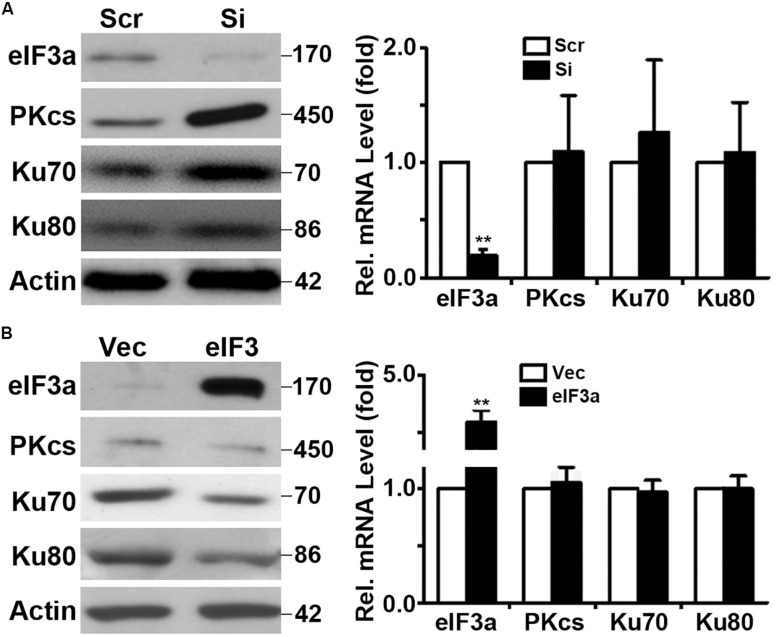
Effect of eukaryotic initiation factor (eIF)3a on expression of genes encoding proteins important for non-homologous end joining (NHEJ) repair. Western blot and real-time reverse transcription (RT)-PCR analyses were performed to determine the effect of eIF3a on the expression of Ku70, Ku80, and DNA-PKcs genes in H1299 cells with transient knockdown **(A)** and NIH3T3 cells with stable eIF3a overexpression **(B)** compared with their respective control cells (*n* = 3; **P < 0.01).

Because eIF3a is known to regulate the synthesis of proteins, we hypothesized that eIF3a may regulate NHEJ repair of DSBs by regulating the synthesis of NHEJ repair proteins. To test this hypothesis, we first performed a Western blot analysis of major proteins involved in NHEJ repair in H1299/Si vs. H1299/Scr and NIH3T3/eIF3a vs. NIH3T3/Vec cells. As shown in Figures 4A,B, the expression of DNA-PKcs, Ku70, and Ku80 in H1299/Si cells was drastically increased compared with the control H1299/Scr cells. Consistently, the expression of these genes in NIH3T3/eIF3a cells was dramatically reduced compared with the control NIH3T3/Vec cells. Interestingly, real-time reverse transcription (RT)-PCR analyses showed no change in the mRNA level of these genes, suggesting that the effect of eIF3a on the expression of DNA-PKcs, Ku70, and Ku80 is likely at the protein, not mRNA, level.

**FIGURE 5:**
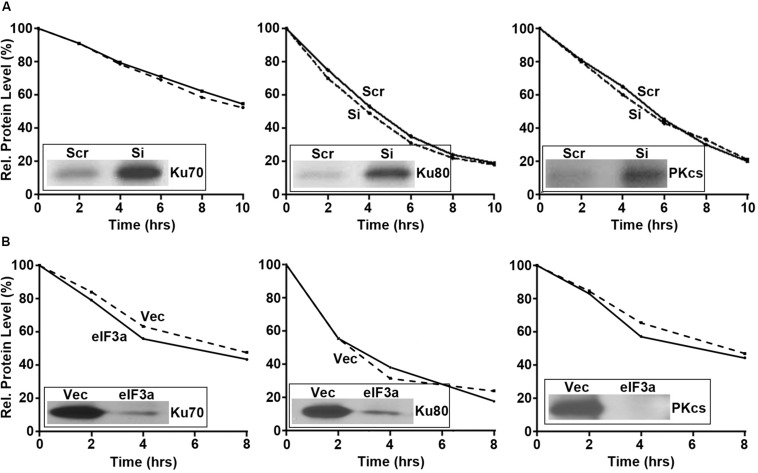
Effect of eukaryotic initiation factor (eIF)3a on the synthesis and degradation of Ku70, K80, and DNA-PKcs. Pulse labeling in combination with immunoprecipitation and cycloheximide chasing were performed to determine the synthesis (insets) and degradation, respectively, of Ku70, Ku80, and DNA-PKcs in H1299 cells with transient eIF3a knockdown **(A)** and NIH3T3 cells with stable eIF3a overexpression **(B)** compared with their respective control cells.

Next, we performed pulse labeling and cycloheximide chase experiments to determine the eIF3a effect on the synthesis and degradation of these DNA repair proteins, respectively. As shown in Figure 5, eIF3a knockdown using siRNA in H1299 cells (Figure 5A) or eIF3a overexpression in NIH3T3 cells (Figure 5B) had no effects on the decay of these DNA repair proteins. However, the synthesis of Ku70, Ku80, and DNA-PKcs was dramatically increased in H1299/Si (Figure 5A) and reduced in NIH3T3/eIF3a (Figure 5B) cells compared with their respective control H1299/Scr and NIH3T3/Vec cells. Thus, eIF3a likely inhibits the synthesis of Ku70, Ku80, and DNA-PKcs proteins, leading to a reduced repair of DSB by the NHEJ pathway.

### High Eukaryotic Initiation Factor 3a Expression Benefits Overall Survival of Cancer Patients

**FIGURE 6:**
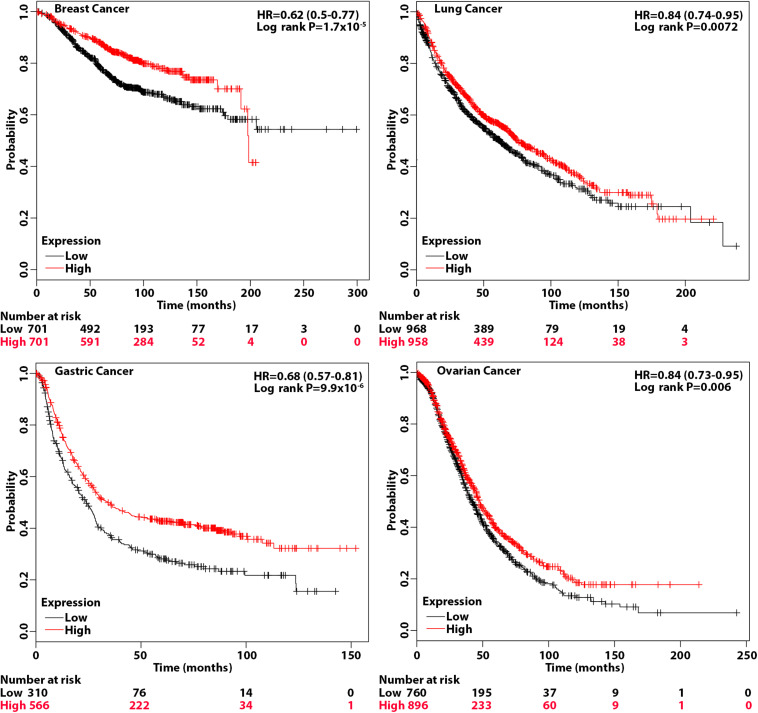
Association of eukaryotic initiation factor (eIF)3a expression with overall survival of breast, gastric, lung, and ovarian cancers. Overall survival analyses were conducted using Kaplan–Meier (KM) plotter with hazard ratio (HR) at 95% confidence intervals and log rank *P* < 0.05 considered significant.

The above studies suggest that patients with tumors that have a high level of eIF3a may be more sensitive to treatments that cause DSBs such as radiotherapy and chemotherapy with drugs that cause DSBs. To test this hypothesis, we performed overall survival analysis of breast, gastric, lung, and ovarian cancer patients using information freely available in Kaplan–Meier (KM) plotter. As shown in Figure 6, breast, gastric, lung, and ovarian cancer patients with a high eIF3a expression level all had better overall survival than patients with a low eIF3a level. These findings are consistent with the eIF3a function in sensitizing cancer cells to treatment that cause DNA damages.

## Discussion

Appropriate combinations of radiation with chemotherapeutic drugs and radiation have resulted in remarkable outcome in cancer treatments. However, resistance to both radiation and anticancer drugs frequently occurs, causing failure in the successful treatment or cure of human cancers. In this study, using cell line models, we show that eIF3a may play important roles in the cellular response to IR with several lines of evidence. Alteration of eIF3a expression not only changes cellular survival following IR treatment, it also affects the levels of DSBs as measured using the comet assay and detecting γ-H2AX, an indicator of DNA damage as well as HCR assay for NHEJ repair activities. Furthermore, eIF3a may regulate the synthesis of DNA repair proteins, Ku70, Ku80, and DNA-PKcs, important for the NHEJ repair pathway. These findings are consistent with the observation that lower eIF3a expression associates with poor prognosis of several cancers in this study and as reported in previous publications (Bachmann et al., 1997; Dellas et al., 1998; Chen and Burger, 1999, 2004; Pincheira et al., 2001; Spilka et al., 2014).

The difference in γ-H2AX protein level between cells with different eIF3a levels was observed early (20 min) following IR treatments. Although eIF3a may sensitize cells from IR-induced DNA damage to reduce the production of γ-H2AX, it is more likely that cells with different eIF3a levels have similar levels of DSBs induced by IR, but the cells with less eIF3a can repair the DSBs much more quickly than the cells with a high eIF3a level, causing the different levels of γ-H2AX in these cells. This difference in repair activity may be due to the difference in the basal level of DNA repair proteins between these cells under different levels of eIF3a regulation. It is noteworthy that DNA-PKcs, the downstream target of eIF3a shown here, has been suggested to phosphorylate H2AX, leading to the production of IR-induced γ-H2AX (Stiff et al., 2004). In this study, although eIF3a knockdown increased the level of DNA-PKcs protein, there is no increase in the basal level of γ-H2AX. In fact, γ-H2AX decreased in eIF3a knockdown cells at 20 min following IR treatment. While this finding is consistent with less DNA damage in eIF3a knockdown cells presumably due to increased NHEJ repair activity, it is inconsistent with possible DNA-PKcs phosphorylation of H2AX. However, because half maximum accumulation of γ-H2AX occurs in 1 min following IR (Rogakou et al., 1998), it is possible that we were unable to detect the IR-induced γ-H2AX increase due to high DNA-PKcs expression with eIF3a knockdown. At 20 min or later after IR when samples were collected, γ-H2AX may have already been reduced due to rapid reduction in DSBs. Future studies are required to test this possibility.

**FIGURE 7:**
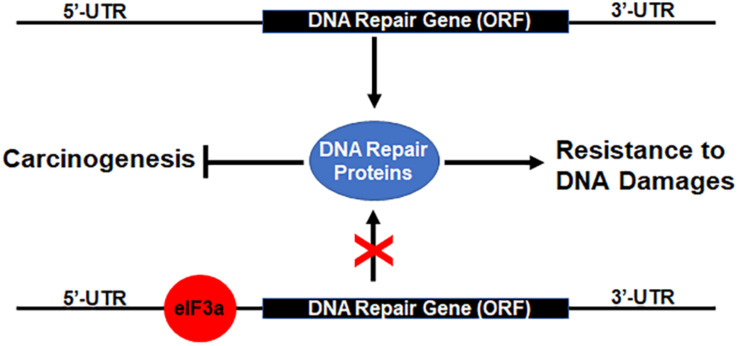
Hypothetical model of eukaryotic initiation factor (eIF)3a regulation of DNA repair protein synthesis relationship with DNA damage resistance and tumorigenesis. Binding of eIF3a to 5′-untranslated regions (5′-UTRs) of mRNAs encoding DNA damage repair proteins inhibits translation and synthesis of DNA repair proteins, leading to sensitization of cancer cells to DNA-damaging treatments and susceptibility of normal cells to DNA-damaging carcinogens.

Previously, it has been shown that eIF3a regulates the synthesis of proteins important for NER and cellular response to cisplatin and that eIF3a may bind to the 5′-untranslated region (5′-UTR) of mRNAs encoding DNA repair proteins and, thus, inhibiting translation of these mRNAs to synthesize these proteins (Liu et al., 2011; Yin et al., 2011b, 2013). Together with these previous findings, our current results suggest that eIF3a likely plays an important role in regulating the synthesis of DNA repair proteins that contributes to cellular response to DNA-damaging drugs or radiation (Figure 7). In a previous study, we showed that eIF3a knockdown sensitized cancer cells to cisplatin-induced apoptosis (Liu et al., 2011), further supporting the conclusion that eIF3a may contribute to cellular sensitivity to DNA-damaging treatments by suppressing DNA repair and by increasing DNA damage-induced apoptosis. We have also found in the previous study (Liu et al., 2011) that a selected cisplatin-sensitive cell line expressed a higher level of eIF3a compared with the parent cell line, suggesting that cancer cells may alter eIF3a expression to adapt to a DNA-damaging environment by regulating DNA damage repair protein synthesis.

As discussed above, eIF3a may also have oncogenic functions (Dong et al., 2004; Zhang et al., 2007). This oncogenic function may relate to the finding that eIF3a suppresses DNA repair *via* suppressing the synthesis of DNA repair proteins. Reduced DNA damage repair activity due to eIF3a overexpression in normal cells likely reduces the protection against DNA-damaging carcinogens, leading to an increased possibility of carcinogenesis (Figure 7). Further studies are required to test this theory.

The fact that eIF3a does not contribute to cellular response to non-DNA-damaging drugs such as *vinca* alkaloid (Yin et al., 2011b) suggests that eIF3a regulation of cellular response to DNA-damaging treatments may be a specific event. Whether eIF3a also regulates the synthesis of proteins important for other DNA repair pathways such as HR for repair of DSBs remains to be determined. Nevertheless, the findings that eIF3a may suppress the synthesis of DNA repair proteins and contribute to the increased sensitivity of cancer cells to DNA-damaging treatments suggest that eIF3a may be developed as a biomarker for precision medicine prescription. Patients with a high level of eIF3a may benefit from DNA-damaging drug and radiation treatment, whereas such treatments may not be as much of a benefit for patients with a low level of eIF3a. Indeed, we observed that patients with a high level of eIF3a expression have significantly better overall survival than patients with a low eIF3a level in breast, gastric, lung, and ovarian cancers. Future studies are also warranted to investigate the possibility to target eIF3a regulation of DNA repair protein synthesis to sensitize resistance to DNA-damaging treatments.

In addition to the regulation in the synthesis of DNA repair proteins, eIF3a has also been observed to possibly regulate the synthesis of p27 and ribonucleotide reductase (Dong and Zhang, 2003; Dong et al., 2004). The findings that eIF3a suppresses the synthesis of DNA repair proteins are against the intuition and belief that eIF3a facilitates translational initiation as a subunit of eIF3 complex, and it would increase protein synthesis in general. Because eIF3a is thought to be a subunit of the eIF3 complex consisting of 13 putative subunits, eIF3a regulation of synthesis of DNA repair proteins may be indirect due to the effect of its upregulation or knockdown on the formation of the complex or sub-complexes of other eIF3 subunits. For example, it is noteworthy that eIF3e was discovered as int-6, a site of mouse mammary tumor virus (MMTV) integration (Asano et al., 1998), suggesting a tumor suppressor function. While the role of eIF3e in translational control is not yet established, the studies in yeast support a tumor suppressor role in ameliorating oxidative damage associated with nutritional stress conditions (Udagawa et al., 2008; Nemoto et al., 2010). In mammals, the functional core of eIF3 is defined with eIF3a, b, c, e, f, and h subunits (Masutani et al., 2007), and eIF3a appears to be the docking site for eIF3a:b:i:g sub-complex formation (Zhou et al., 2008; Dong et al., 2013). In light of these previous findings, it is possible that eIF3a knockdown or overexpression changes the spectrum in forming eIF3 sub-complexes including other eIF3 subunits such as eIF3e, which may mediate the regulation of DNA repair protein synthesis.

As an alternative possibility, eIF3a may have an additional regulatory non-canonical function not associated with the eIF3 complex or sub-complexes. The fact that eIF3a suppresses the synthesis of some proteins (e.g., DNA repair proteins) while increasing the synthesis of others (e.g., ribonucleotide reductase) (Dong et al., 2004) and Chk1 (Dong et al., 2020) supports this concept. How eIF3a regulates protein synthesis with its non-canonical activity remains unknown. However, the previous finding that eIF3a can bind to the 5′-UTRs of RPA2 mRNA (Yin et al., 2013) suggests that eIF3a may bind to these mRNAs and suppress the translation of these mRNAs. It also remains to be determined whether this non-canonical activity requires other eIF3 subunits such as eIF3b or eIF3i. Recently, eIF3i has also been shown to be able to regulate the synthesis of cyclooxygenase (COX)2 (Qi et al., 2014), suggesting that other eIF3 subunits may also have non-canonical activity, although it is not clear if they work together. Clearly, more studies are needed to investigate further the non-canonical function of eIF3 subunits vs. alteration of eIF3 complex/sub-complex formation in regulating protein synthesis.

## Materials and Methods

### Materials

Cell culture media and reagents were purchased from BioSources International (Camarillo, CA), Media Tech (Herndon, CA), or Cambrex (Walkersville, MD). All electrophoresis reagents and polyvinylidene difluoride (PVDF) membranes were purchased from Bio-Rad (Hercules, CA). SYBR Green PCR Master Mix for real-time PCR, the high-capacity cDNA reverse transcription kit, and all primers were purchased from Applied Biosystems-Thermo Fisher (Chicago, IL). Metafectene^®^ pro was purchased from Biontex (San Diego, CA). All other chemicals and reagents were of molecular biology grade from Sigma (St. Louis, MO) or Fisher Scientific (Chicago, IL).

### Cell Culture and Survival Assay

Human lung cancer cell line H1299 and NIH3T3 cells were cultured at 37°C with 5% CO_2_ in RPMI 1640 and Dulbecco’s modified Eagle’s medium (DMEM) medium, respectively, supplemented with 10% fetal bovine serum, 100 units/ml penicillin, and 100 μg/ml streptomycin. G418 at 400 μg/ml was also supplemented in the culture of the stable NIH3T3 cells overexpressing eIF3a and vector-transfected controls, which were established in our previous study (Yin et al., 2011b).

Colony formation survival assay was performed following IR treatments as previously described (Li et al., 2010; Wu et al., 2016). Briefly, 100 cells/well were seeded in six-well plates and cultured for 24 h followed by treatments with different doses of IR and continuous culture for 10–14 days with media changed every 2–3 days. At the end of the study, cells were washed with phosphate-buffered saline (PBS), and the colonies were stained with 0.05% (w/v) crystal violet in PBS containing 20% methanol for 15 min at room temperature and counted manually.

### Western Blot, Immunofluorescence Staining, and Immunoprecipitation

Western blot analysis and immunofluorescence staining were performed as previously described (Dong and Zhang, 2003; Wu et al., 2016). Briefly, for Western blot analysis, equal amount of proteins from different cells were separated by sodium dodecyl sulfate–polyacrylamide gel electrophoresis (SDS-PAGE), transferred to PVDF membranes, and probed with antibodies specific to eIF3a, γ-H2AX, Ku70, Ku80, DNA-PKcs, and actin control followed by probing with horseradish peroxidase (HRP)-conjugated secondary antibody and enhanced chemiluminescence (ECL) reagents. The signals are captured on X-ray films.

Immunofluorescence staining was performed by culturing cells on glass coverslips, which were washed twice with cold PBS and fixed with acetone/methanol mixture (1:1) for 10 min and blocked in 1% bovine serum albumin (BSA) and 1% normal horse serum in PBS at 4°C for 30 min. The coverslips were then probed with the γ-H2AX-specific antibody for 30 min at 4°C followed by incubation with fluorescein isothiocyanate (FITC)-conjugated secondary antibody at room temperature for 30 min and washed twice with PBS. The cells were counterstained with 4′,6-diamidino-2-phenylindole (DAPI) before viewing on a confocal microscope.

Immunoprecipitation was performed as previously described (Dong and Zhang, 2003). Briefly, cell lysates were first mixed with mouse immunoglobulin G (IgG) and incubated for 2 h at 4°C followed by addition of 50% protein G-agarose slurry and incubation for 3 h at 4°C to remove non-specifically bound proteins by centrifugation. The supernatant was transferred to a new tube and incubated with 5 μg primary antibodies against Ku70, Ku80, or DNA-PKcs at 4°C for 2 h. Finally, 50 μl 50% protein G-agarose beads was added to the mixtures and incubated at 4°C overnight. The immunoprecipitated materials were collected by centrifugation and extensive washing with lysis buffer followed by separation using SDS-PAGE.

### Real-Time Reverse Transcription-PCR

Real-time RT-PCR was performed as described previously (Dong et al., 2004, 2005). Briefly, total RNA was extracted using RNeasy Mini Kit (Qiagen, Valencia, CA), and real-time RT-PCR was performed using Power SYBR Green RNA-to-CT 1-Step kit. Data were normalized to an internal control gene, glyceraldehyde-3-phosphate dehydrogenase (GAPDH). The sequences of primers for the PCRs were 5-CATGGCAACTCCAGAGCAG (forward) and GCTCCTTAAACTCATCCACC (reverse) for Ku70; AGAAGAAGGCCAGCTTTGAG (forward) and AGCTGTGACAGAACTTCCAG (reverse) for Ku80; CCGGACGGACCTACTACGACT (forward) and AGAACGACCTGGGCATCCT (reverse) for DNA-PKcs.

### Comet Assay

Neutral comet assay was performed as previously described (Wu et al., 2016). Briefly, cells treated with or without IR were embedded in low melting agarose on a microscope slide followed by lysis at neutral pH and electrophoresis in Tris-borate-ethylenediaminetetraacetic acid (TBE) buffer for 2 h. Comets were observed after staining the cells with SYBR Green I and scoring 120 cells in each sample to measure the Olive tail moment.

### Host Cell Reactivation-Based Non-homologous End Joining Assay

Host cell reactivation NHEJ assay was performed using pGL3 reporter plasmid with firefly luciferase gene driven by cytomegalovirus (CMV) promoter as previously described (Wu et al., 2016). Briefly, pGL3 was first linearized by *Hin*dIII digestion and co-transfected into cells along with the control circular pRL-TK reporter plasmid encoding Renilla luciferase followed by determination of both firefly and Renilla luciferase activity. Expression of firefly luciferase is dependent on the repair of the plasmid to regenerate the circular plasmid *via* NHEJ.

### Protein Synthesis and Stability Assays

Pulse labeling using [^35^S]methionine in combination with immunoprecipitation and SDS-PAGE analysis was used to determine the protein synthesis rate as previously described (Dong and Zhang, 2003; Dong et al., 2004). Briefly, cells were washed with PBS and methionine-free media before being subjected to labeling with [^35^S]methionine (10 μCi/ml) in methionine-free media for 2 h. The cells were then washed with PBS and harvested for cell lysate preparation, immunoprecipitation, and separation by SDS-PAGE. The signals for immunoprecipitated proteins labeled by [^35^S]methionine were captured by exposing to X-ray films.

The stability of specific proteins was determined using cycloheximide chasing as previously described (Liu et al., 2006). Briefly, cells were treated with 10 μg/ml cycloheximide for different times and harvested for preparation of cell lysates and Western blot analyses. The quantity of each protein at each time point of cycloheximide treatment was determined using the ImageJ software (NIH, USA) and plotted against the treatment time.

### Overall Survival Analyses

The KM plotter can assess the effect of 54,675 genes on survival using 10,461 cancer samples, including 5,143 breast, 1,816 ovarian, 2,437 lung, and 1,065 gastric cancer patients with a mean follow-up of 69/40/49/33 months (Lanczky et al., 2016). The analyses on all four cancers were conducted using default parameters, and the hazard ratio (HR) with 95% confidence intervals and log rank *P* < 0.05 considered significant were calculated and shown on the plot.

## Data Availability Statement

Publicly available datasets were analyzed in this study. This data can be found here: http://kmplot.com/analysis.

## Author Contributions

RT performed the experiments and helped to draft the manuscript. CW, TD, JC, JB, SM, and ZD performed the experiments. J-YL performed the data analysis. J-TZ designed the studies, wrote the manuscript, and provided the support. All authors contributed to the article and approved the submitted version.

## Conflict of Interest

The authors declare that the research was conducted in the absence of any commercial or financial relationships that could be construed as a potential conflict of interest.
