# Corrigendum: eIF3a Regulation of NHEJ Repair Protein Synthesis and Cellular Response to Ionizing Radiation

**DOI:** 10.3389/fcell.2020.629218

**Published:** 2021-01-07

**Authors:** Rima Tumia, Chao J. Wang, Tianhan Dong, Shijie Ma, Jenny Beebe, Juan Chen, Zizheng Dong, Jing-Yuan Liu, Jian-Ting Zhang

**Affiliations:** ^1^Department of Pharmacology and Toxicology, Indiana University School of Medicine, Indianapolis, IN, United States; ^2^Department of Cancer Biology, University of Toledo College of Medicine and Life Sciences, Toledo, OH, United States; ^3^Department of Medicine, University of Toledo College of Medicine and Life Sciences, Toledo, OH, United States

**Keywords:** eukaryotic initiation factor 3a (eIF3a), DNA repair, radiation, resistance, mRNA translation, protein synthesis, gamma-H2A histone family member X (γ-H2AX)

In the original article, there was a mistake in Figure 1A and Figure 3A as published. Wrong images for the Western blot of H1299 cells in Figure 1A and for the comet assay of the control un-irradiated H1299 cells in Figure 3A were accidently used for publication. The corrected Figures 1 and 3 appear below.

**Figure 1:**
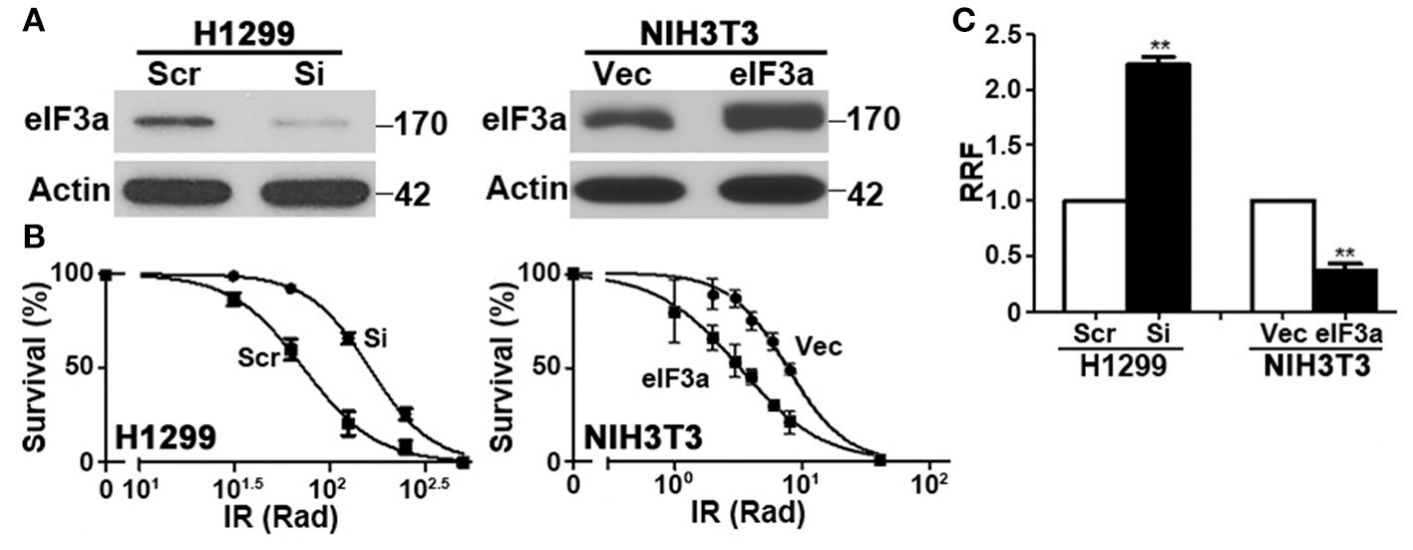
Effect of eukaryotic initiation factor (eIF)3a expression on the cellular response to ionizing radiation (IR). Western blot analyses **(A)** and colony formation assay following IR treatment **(B)** of H1299 cells with transient eIF3a knockdown and NIH3T3 cells with stable eIF3a overexpression compared with their respective control cells. Actin was used as a loading control. Panel **(C)** shows a summary of eIF3a effects on cellular sensitivity to IR treatments. Relative resistance factor (RRF) was derived by dividing the IC_50_ of the test cells by that of their control cells (*n* = 3, ^**^P < 0.01).

**Figure 3:**
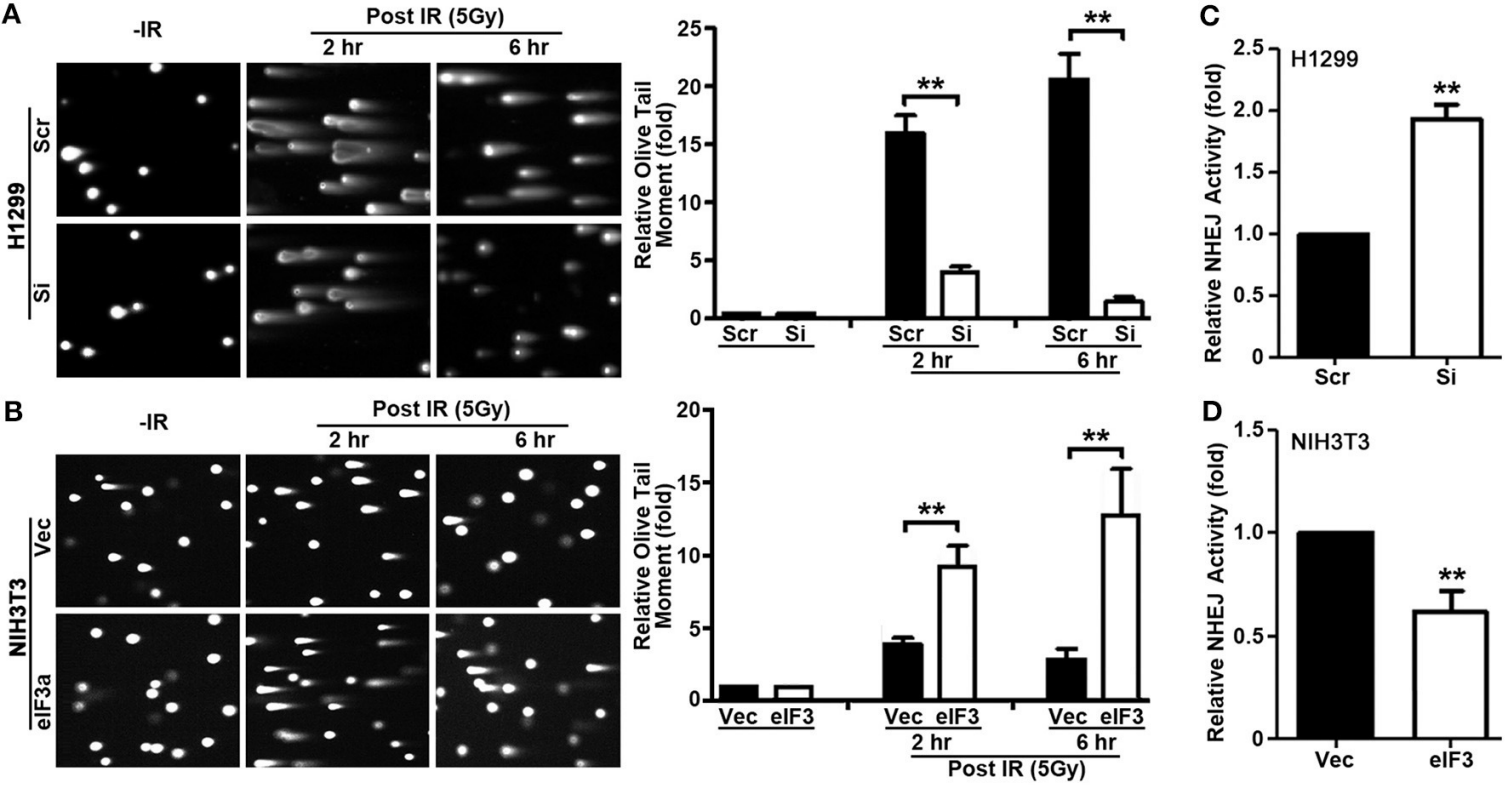
Role of eukaryotic initiation factor (eIF)3a in non-homologous end joining (NHEJ) repair of ionizing radiation (IR)-induced double-strand breaks (DSBs). **(A,B)** Comet assay was used to determine eIF3a effects on the level of DSBs induced by IR in H1299 cells with transient eIF3a knockdown **(A)** and NIH3T3 cells with stable eIF3a overexpression **(B)** compared with their respective control cells. The histograms show the summary of quantitative analysis of Olive tail moment in these cells. **(C,D)** Host cell reactivation assays using reporter constructs were performed using H1299 cells with eIF3a knockdown **(C)** and NIH3T3 cells with eIF3a stable overexpression **(D)** compared with their respective control cells (*n* = 3; ^**^P < 0.01).

The authors apologize for this error and state that this does not change the scientific conclusions of the article in any way. The original article has been updated.

